# Molecular characterization and expression dynamics of MTP genes under various spatio-temporal stages and metal stress conditions in rice

**DOI:** 10.1371/journal.pone.0217360

**Published:** 2019-05-28

**Authors:** Hasthi Ram, Amandeep Kaur, Nishu Gandass, Shweta Singh, Rupesh Deshmukh, Humira Sonah, Tilak Raj Sharma

**Affiliations:** Department of Agri-Biotechnology, National Agri-Food Biotechnology Institute (NABI), SAS Nagar(Mohali), Punjab, India; National Institute for Plant Genome Research, INDIA

## Abstract

Metal Tolerance Proteins (MTPs) are the class of membrane proteins involved in the transport of metals, mainly Zn, Mn, Fe, Cd, Co and Ni, and confer metal tolerance in plants. In the present study, a comprehensive molecular analysis of rice MTP genes was performed to understand the evolution, distribution and expression dynamics of MTP genes. Exploration of the whole genome re-sequencing information available for three thousand rice genotypes highlighted the evolution and allelic diversity of MTP genes. Based on the presence of non-synonymous single nucleotide polymorphism (SNP), MTP1, MTP6, MTP8 and MTP9 were found to be the most conserved genes. Furthermore, results showed localization of MTP1, MTP8.1 and MTP9, and MTP11, respectively with QTLs/m-QTLs for Zn and Cd accumulation, making these genes promising candidates to understand the QTL regulation. Expression profiling of the entire set of 10 MTP genes revealed root and shoot specific expressions of MTP9 and MTP8.1, respectively, under all tested vegetative stages. Expression of seed-specific MTPs increased as seed maturation progressed, which revealed their potential role in transporting metals during seed filling. Upon exposure to harmful heavy metals, expression of most MTP genes decreased in root and increased in shoot, suggests that different mechanisms are being employed by MTPs in different tissues. Contrastingly, only a few MTPs were found to be responsive to Fe and/or Zn starvation conditions. The extensive analysis of MTPs presented here will be helpful in identifying candidate MTP genes for crop biofortification and bioremediation purposes.

## Introduction

Plants, being sessile, are continuously exposed to several biotic and abiotic stresses, including nutritional constraints. Among the abiotic stresses, heavy metal stresses imposed by many contaminants present in the environment adversely affect plant growth [1]. Some heavy metals, such as Zn and Fe, play an important role in the physiological and biochemical processes of the plants [2,3]. However, at higher concentrations they can also cause toxicity, therefore, their uptake and utilization are tightly controlled in the plants [4,5]. Micronutrient deficiency, especially deficiency of essential metals such as Zn and Fe in humans, is considered a major health challenge in developing countries [6]. Some other heavy metals, such as Cd, Cr, Pb, Hg, and Co, are non-essential for both plants and humans and are very toxic even at very low concentrations [7,8]. Hence, many efforts are being made to increase the amount of essential metals such as Zn and Fe, and restrict the accumulation of non-essential toxic heavy metals in edible organs of plants [9]. Some plants, known as hyperaccumulators, can uptake large amounts of heavy metals from the soil and store in their aboveground organs [10], which makes these plants ideal to be utilized for bioremediation purposes to remove heavy metals from the soil. Knowledge gained from studies related to distribution of heavy metals in plants is extremely helpful for the efforts employed towards biofortification and bioremediation.

Cation Diffusion Facilitator (CDF) family genes are integral membrane divalent cation transporters that either transport metal ions out of the cytoplasm into extracellular space or into the vacuoles]. Members of the CDF family have been identified in prokaryotes, eukaryotes, archaea and plants, suggesting that they are evolutionary conserved [11]. Most of the CDF proteins possess six transmembrane domains (TMDs) and a conserved C-terminal domain protruding into the cytoplasm [12]. Based on the substrate specificity evaluated for some members, the CDF genes are categorised into three major families, namely, Zn-CDFs, Zn/Fe-CDFs, and Mn-CDFs [13]. In plants, the CDFs are called Metal Tolerance Proteins (MTPs)]. The plant MTPs are grouped into seven groups, namely group 1, 5, 6, 7, 8, 9, 12, and the group nomenclature was done based on annotated MTP sequences from Arabidopsis [12]. Of the ten rice MTP genes, seven (MTP1, MTP5, MTP6, MTP7, MTP8, MTP9, and MTP12) were assigned to their respective number group (group no. 1, 5, 6, 7, 8, 9 and 12) and of the remaining, MTP8.1 was assigned to group 8, and MTP11 and MTP11.1 were assigned to group 9 [12]. Group 8 and 9 rice MTPs (MTP8, MTP8.1, MTP9, MTP11, MTP11.1) belong to the Mn-CDF family, MTP6 and MTP7 belong to the Zn/Fe-CDF family, and MTP1, MTP5 and MTP12 belong to Zn-CDF family [14,15]. Functional analysis of some of the Mn-CDF family genes revealed that these genes specifically mediate Mn tolerance through sequestration of Mn into the shoot and root vacuoles, and they are not involved in tolerance against Fe, Zn, Co, Ni or Cd [14,15].

Previous studies on rice and other plant species have mostly evaluated individual MTP for understanding of substrate specificity and transport [16]. However, very little efforts have been made towards the understanding of regulation of entire MTP family under different heavy metal conditions, tissues and developmental stages. It is not yet clear which MTP gene is most important for tolerance against particular heavy metal. Furthermore, it is also not clear whether specific MTP plays role in specific tissues such as in root, shoots or grain. In this regard, understanding of MTP gene expression dynamics under the condition with heavy metals exposure or starvation conditions of essential minerals would be helpful to close these gaps in our understanding. In this regard, we have performed the gene expression profiling of all the rice MTP genes across various stages of vegetative and reproductive growth to identify the stage specific MTP genes in Indica rice variety IR64, which provides very high yield, early maturity, disease resistance and excellent cooking quality [17].Furthermore, to understand the role of MTP genes against specific heavy metal species, we have analyzed the response of all the rice MTP genes to various heavy metal conditions. Finally, from a biofortification perspective, the response of each MTP gene to Fe and Zn starvation conditions was also studied. The detailed analysis about evolution, distribution, and expression dynamics of MTP genes in rice can be explored for biofortification as well as bioremediation purposes.

## Materials and methods

### Identification and structural organization of MTP genes

In this study, search was performed for term ‘Metal Tolerance Protein’ at RAP database and Oryzabase, we found the rice genes which are annotated as Metal Tolerance Proteins. To further identify new MTP genes in the rice genome, we used OsMTP1 amino acid sequences as a query to perform a database search using BLASTP against predicted proteins in the Oryza *sativa* genome derived from the RAP database. BLAST hits with less than 100 bitscore were removed. With these criteria, we didn’t find any new MTP genes in the rice genome. Genomic DNA sequences, CDS and protein sequences for all the genes, except for MTP12, were retrieved from the RAP Database. The exon/intron organization of MTP was visualized with the Gene Structure Display Server (GSDS) program (http://gsds.cbi.pku.edu.cn/).

### Phylogenetic and evolutionary analysis of MTP genes

Multiple sequence alignments were conducted with amino acid sequences. A phylogenetic tree was constructed using the Maximum-likelihood method (MLM) provided in the MEGA 7.0 software tool. Robustness of the branch nodes was confirmed with bootstrap analysis performed with 10000 replications. To study the molecular evolution of MTP genes, the publically available SNP dataset generated from the whole genome resequencing of 3000 rice genotypes was used [18]. All the SNP data for ten MTP genes were converted into hapmap format and diversity analysis was performed using Tassel V5.0 software (http://www.maizegenetics.net/tassel). Functional annotation of SNPs was performed using SnpEff tool, and the effect of the amino acid variation was predicted using PROVEAN tool [19]. PROVEAN scores equal to or below -2.5 were considered deleterious, and the score above -2.5 was considered neutral.

### Identification of conserved motifs, subcellular location and structural prediction

The protein sequences were analyzed to identify conserved protein motifs (motif scan) using the MEME (multiple EM for motif elicitation) program. Transmembrane domains in MTP proteins were identified using TOPCONS software tools. Subcellular localization of MTP genes was performed using CELLO server (http://cello.life.nctu.edu.tw/). The protein sequences of all MTP proteins were submitted to the Phyre2 protein-modeling server (www.sbg.bio.ic.ac.uk/*phyre2). The protein 3D structure were further refined using ModRefiner(https://zhanglab.ccmb.med.umich.edu/ModRefiner/).

### Construction of hierarchical clustering and heatmap of expression data

The heatmap of log2 transformed values of MTP genes was generated using MeV4.9 software tool (http://mev.tm4.org/). The hierarchical clustering was performed using HCL option in MeV software tool with covariance values for distance metric selection. The average linkage clustering method was selected for clustering.

### Plant growth and treatment conditions

*Oryza sativa* subspecies *indica* cultivar *IR-64* was used for all the experiments. To study gene expression, seeds were surface-sterilized with 1.2% sodium hypochlorite in 10% ethanol, followed by washing with double autoclaved water, and kept overnight on moist Whatman filter paper in a Petri dish at 4°C for stratification. The next day seeds were transferred to a plant growth chamber (Conviron, Canada) under a 16 h photoperiod at 400 μmolm^-2^s^-1^, with 70% relative humidity and temperature 28°C/25°C (day/night). After 3–4 days of growth, the seedlings were transferred to hydroponics conditions in plastic boxes containing soft foam pieces (to hold the roots) and filled with Yoshida Nutrient Solution (YNS). The composition of modified YNS is as follows: 1.77 mM NH_4_NO_3_, 0.32 mM NaH_2_PO_4_.2H_2_O, 0.5 mM K_2_SO_4_, 1 mM CaCl_2_.2H_2_O, 1 mM MgSO_4_.7H_2_O, 9 μM MnCl_2_.4H_2_O, 0.5 μM (NH_4_)6Mo_7_O_24_.4H_2_O, 18.5 μM H_3_BO_3_, 0.16 μM CuSO_4_.5H2O, 5 um ZnSO_4_, 36 μM FeNaEDTA. After one week of growth after germination, seedlings were subsequently transferred to YNS with no FeNaEDTA (Fe-) or no ZnSO_4_ (Zn-), or 50 μM NiCl_2_ (Ni+) or 50um CoCl_2_ (Co+) or 100um CdCl_2_ (Cd+) for different time points. For Fe- and Zn- conditions, tissues were harvested after 1 week and 2 week of treatment, whereas for Ni+, Co+ and Cd+ tissues were harvested after 24 hr and 48 hr after treatment. Untreated samples, which were continuously grown in YNS, were used as a control for comparison during the course for all the experiments. After the treatment was over, seedlings were removed from the YNS and roots were dried on paper towels and roots and shoots were then separated and immediately snap frozen in liquid nitrogen and stored at −80°C until further use. Three biological replicates were collected for each condition. To collect seed tissues at different stages, plants were grown in NABI field at Mohali main campus during the summer of 2018 in fertile soil. Seeds at different days after flowering time points were collected and immediately snap frozen in liquid nitrogen and stored at −80°C until further use.

### RNA extraction and cDNA synthesis

Total RNA was extracted from different samples using TRI Reagent (Sigma), as per the supplier’s protocol. Briefly, after powdering the sample with pestle-mortar, the sample was homogenized in Tri Reagent. The extract was mixed with chloroform and centrifuged, and the upper aqueous phase was mixed with 2-propanol to pellet RNA. Afterwards the RNA pellet was washed twice with 70% ethanol. The concentration and RNA quality were checked on Nanodrop (Thermo Scientific) and by agrose gel electrophoresis. Before proceeding to cDNA synthesis RNA was treated with DNase I (RNase free) (New England Biolabs) to remove DNA contamination. After that, DNase was heat inactivated by incubating at 75°C. First strand cDNA was synthesized using 1μg total RNA using iScript-cDNA synthesis kit (Bio-rad Inc.) as per the manufacturer’s instructions.

### Q-PCR analysis

Quantitative real-time PCR was performed using 5× diluted cDNAs and iTaq Universal SYBR Green Supermix (Bio-rad Inc.) on CFX96 Touch Real-Time PCR machine (Bio-rad Inc.) In 10 μl reaction, 5 μl 2x SYBR Green Supermix, 0.5 μl 10 μM primers (Forward + Reverse), 1 μl diluted cDNA, and remaining amount of water was used. Rice *UBQ5* and *eEF-1a* were used as internal reference genes for seedling and seed tissues, respectively. The sequence of the primers used for Q-PCR analysis is provided in S2 Table. Delta-delta CT-method (2−ΔΔCT) was used to calculate relative gene expression levels [20]. The statistical significance of expression data was determined using two-tailed paired Student’s t test.

## Results

### Identification, phylogenetic relationship, gene structure, protein structure and molecular evolution of MTP genes in the rice genome

A total of 10 MTP genes were identified consistently in both the rice sub-species, *indica* and *japonica*. The DNA sequence details for the rice MTP genes are summarized in Table 1.

**Table 1:** Details of the metal tolerance protein (MTP) genes identified in rice genome.

S. No.	Gene name	Locus ID^1^	NCBI REFSEQ Accession no. (gene ID)	Chr. No.	Gene length (bp)	cDNA length (bp)	No. of introns	Protein length (aa)	Protein Mol. Wt. (kDa)
1	*OsMTP1*	Os05g03780	XM_015784798.1, XM_015784797.1 (4337694)	5	3560	1811	1	419	45.579
2	*OsMTP5*	Os02g58580	XM_015771775.1, XM_015771776.1 (4330561)	2	5294	1324	7	277	30.108
3	*OsMTP6*	Os03g22550	XM_015773216.1 (4332819)	3	4995	1671	11	510	55.374
4	*OsMTP7*	Os04g23180	XM_015781753.1 (9269174)	4	6312	1749	12	473	51.329
5	*OsMTP8*	Os02g53490	XM_015771721.1 (4330894)	2	2341	1453	6	411	45.732
6	*OsMTP8*.*1*	Os03g12530	XM_015777805.1 (4332125)	3	4876	2104	6	398	44.704
7	*OsMTP9*	Os01g03914	XM_015765884.1 (432597)	1	3460	1581	5	392	44.793
8	*OsMTP11*	Os01g62070	XM_015757202.1 (4327371)	1	4535	1751	5	416	46.102
9	*OsMTP11*.*1*	Os05g38670	XM_015782691.1 (4339024)	5	3711	1131	5	377	41.907
10	*OsMTP12*	Os08g32650	XM_015795072.1 (4345598)	8	2403	2403	0	801	86.86

^1^Locus ID from Rice Genome Annotation Project with prefix “LOC_”; Mol. Wt.–molecular weight

**Fig 1:**
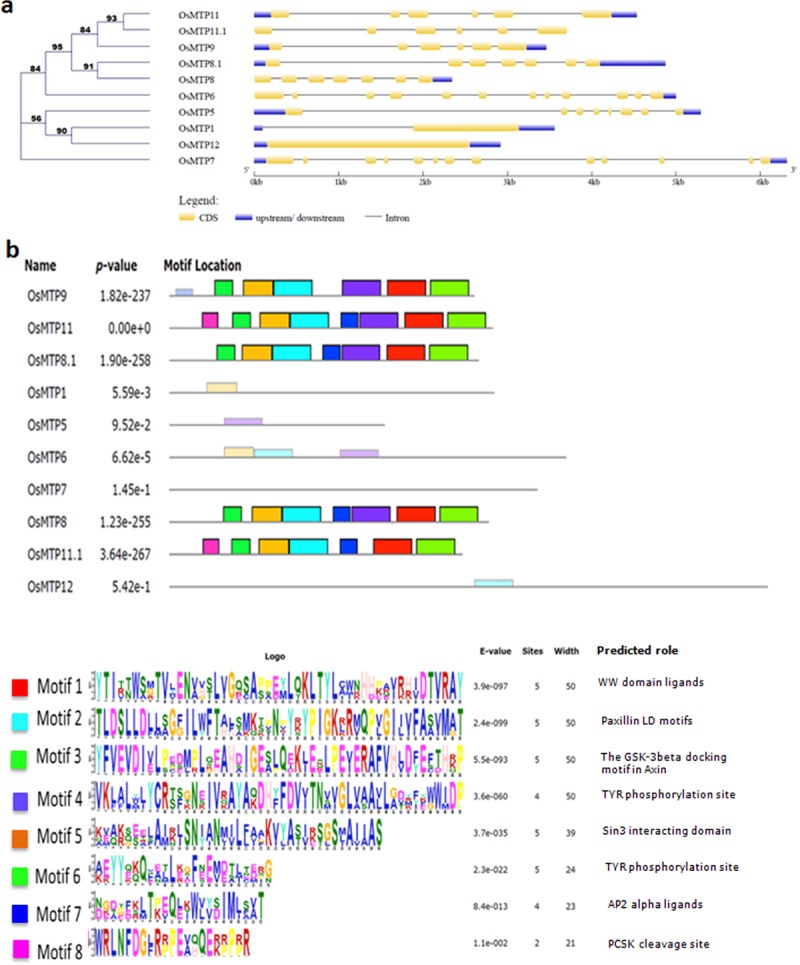
Phylogenetic relationship, exon-intron structure and identification and distribution of conserved motifs of rice metal tolerance proteins (MTPs). (a) The un-rooted phylogenetic tree was constructed via alignment of full-length amino acid sequences from rice using MEGA 7.0 software by using Maximum-likelihood method. Lengths of the exons and introns of each MTP gene are displayed proportionally. Yellow rectangles and thin lines indicate exons and introns, respectively. The un-translated regions (UTRs) are indicated by blue rectangles. (b) All motifs were identified by Multiple EM for Motif Elicitation (MEME) tool using the complete amino acid sequences of OsMTP proteins. Different colored boxes indicate different motifs. Only highlighted motifs are searched by MEME software and the annotation of each motif is listed at the bottom panel. The non-highlighted motifs are searched by other motif finding software.

Phylogenetic tree developed using Maximum-likelihood methods (MLM) showed expectedly a cluster of MTP8, MTP8.1, MTP9, MTP11, and MTP11.1, classified as Mn-CDF, distinct from other MTPs (Fig 1A). Analysis of gene intron-exon organization revealed more conserved gene structure for these Mn-CDF members, with 5–6 introns present in each gene (Fig 1A). Similarly, MTP1, MTP5 and MTP12 classified as Zn-CDFs, were found to be clustered together in the phylogenetic tree. Gene intron-exon organization is also very similar to the Zn-CDF genes, particularly for MTP1 and MTP12, which were found to have a single exon (Fig 1A). The MTP6 and MTP7 respectively having 11 and 12 introns clustered apart from the rest of MTPs in phylogenetic tree (Fig 1A).

Exploration of whole genome re-sequencing data available for three thousand rice genotypes showed high level of genetic diversity for the MTP genes (Table 2). Besides having high frequency of single nucleotide polymorphism (SNP), four MTP genes MTP1, MTP6, MTP8, and MTP9 showed high level of protein sequence conservation (Table 2). Unusually high frequency of non-synonymous SNPs was observed in MTP11.1 and MTP12. Further investigations performed to verify the facts revealed that all six non-synonymous SNPs represent a rare allele present in less than five percent of total re-sequenced genotypes. For both the MTP11.1 and MTP12 genes the minor allele is present exclusively in *indica* lines. Tajima's D, a powerful population genetics test, performed with the SNPs identified within the genic region of MTP genes showed positive Tajima’s D value for all the genes (Table 3). The observed range of Tajima’s D was from 0.3 to 4.8, suggesting a more balancing selection for most of the MTPs.

**Table 2:** Single nucleotide polymorphism (SNP) for metal tolerance protein genes in three thousand rice genotypes.

S. No.	Gene name	Locus ID^1^	Non-syn/syn^2^	Total SNPs
1	OsMTP1	Os05g03780	0/2	17
2	OsMTP5	Os02g58580	1/2	34
3	OsMTP6	Os03g22550	0/2	12
4	OsMTP7	Os04g23180	3/2	61
5	OsMTP8	Os02g53490	0/1	8
6	OsMTP8.1	Os03g12530	4/5	26
7	OsMTP9	Os01g03914	0/7	117
8	OsMTP11	Os01g62070	1/0	30
9	OsMTP11.1	Os05g38670	6/1	29
10	OsMTP12	Os08g32650	6/1	29

^1^Locus ID from Rice Genome Annotation Project with prefix “LOC_”

^**2**^Non-synonymous/synonymous

**Table 3:** Genetic diversity analysis of MTP genes in three thousand rice genotypes.

Gene Name	Chrno.	Start Chr. Position	End Chr. Position	Site Count	Avg. Site Count	Seg Sites	Pi Per BP	Theta Per BP	Tajima’s D
*OsMTP1*	5	1675512	1679044	57	51.13	20	0.117	0.041	3.976
*OsMTP5*	2	35807206	35812237	89	77.96	40	0.157	0.053	4.773
*OsMTP6*	3	12955037	12958282	43	41.37	17	0.080	0.046	1.550
*OsMTP7*	4	13176843	13183039	112	98.60	69	0.170	0.073	3.477
*OsMTP8*	2	32721134	32723722	37	34.52	34	0.122	0.108	0.321
*OsMTP8*.*1*	3	6636642	6641479	47	43.60	31	0.156	0.077	2.396
*OsMTP9*	1	1673117	1680615	476	429.24	152	0.065	0.038	1.953
*OsMTP11*	1	35916773	35921961	72	70.20	35	0.108	0.057	2.161
*OsMTP11*.*1*	5	22670969	22674617	64	59.76	36	0.104	0.066	1.368
*OsMTP12*	8	20219095	20221976	106	97.08	46	0.085	0.051	1.672

**Fig 2:**
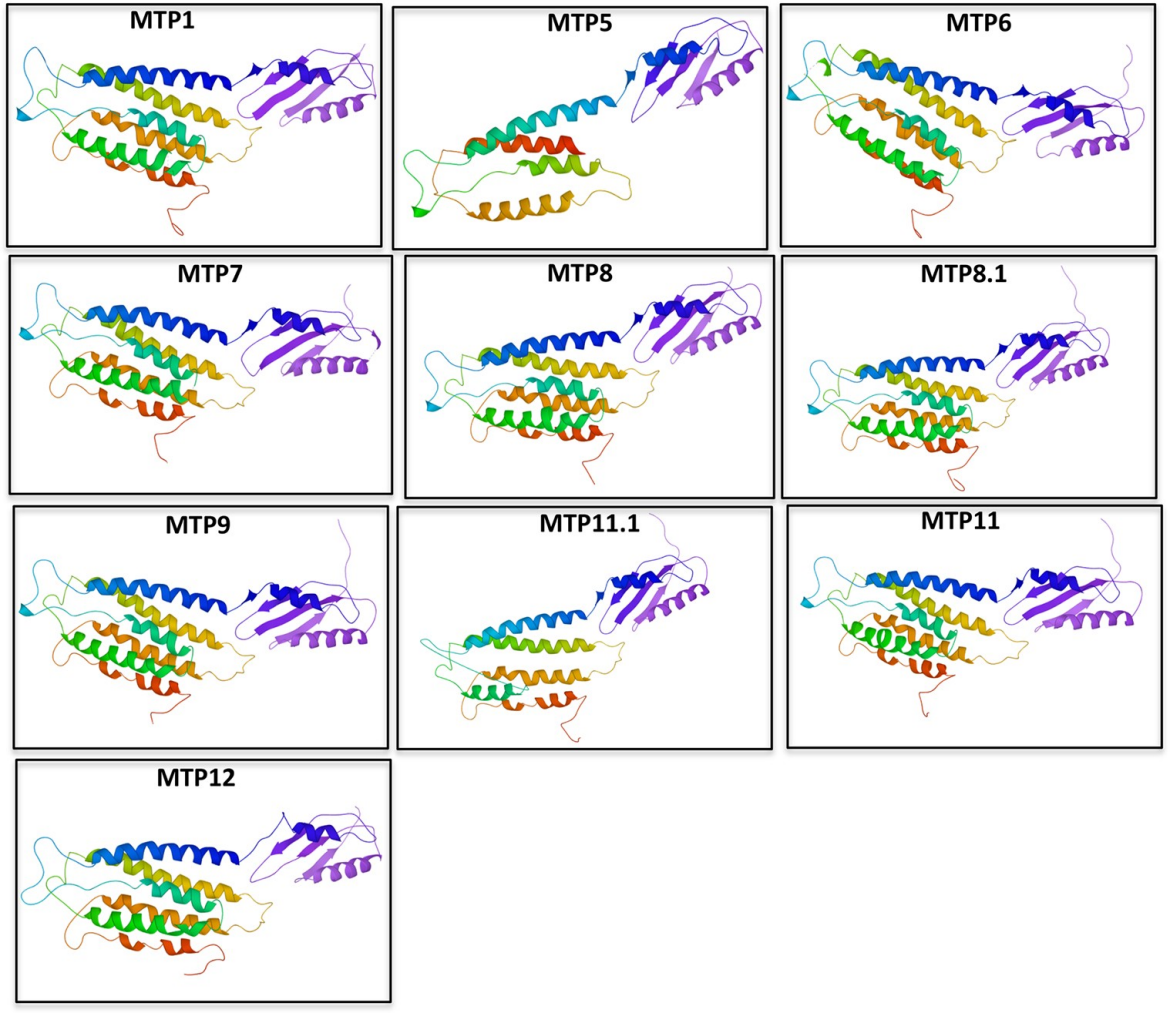
Predicted 3D models of rice MTP proteins. Models were generated by using Phyre2 protein-modeling server at intensive mode. The protein 3D structures were further refined using ModRefiner.

Evaluation of two-dimensional structure predicted using TMHMM tool showed a wide range of transmembrane (TM) domains (S1 Table). Surprisingly, no TM domain was observed in MTP6. To confirm the discrepancies in TM domain prediction, homology based three-dimensional (3D) structures were predicted for the entire set of rice MTP proteins. Unlike TMHMM results, homology based 3D structure showed typical MTP structure with 10 to 12 transmembrane domains for all the rice MTPs including MTP6 (Fig 2). Further search for conserved motifs in rice MTPs was performed with the MEME (multiple EM for motif elicitation) program to gain additional insights into their diversity. As shown in Fig 1B, eight conserved motifs, designated as motif 1 to motif 8, were identified across the MTP family. Interestingly, all the MTPs belonging to Mn-CDF (MTP8, MTP8.1, MTP9, MTP11, MTP11.1) have the same motifs in their protein structure (Fig 1B). However, the remaining MTP proteins didn’t have any of the motifs present in the Mn-CDF family MTPs. Some of these motifs have TYR phosphorylation site, ligand binding domains or have role in cytoskeleton reorganization (Fig 1B)A systematic survey of previously identified QTL and meta-QTLs (m-QTLs) associated with accumulation of metals or tolerance to various metal stress conditions was performed to check whether any of the MTP genes overlap with them (Table 4). Co-localization of MTP8.1 with two m-QTLs, and overlap of MTP1, and MTP9 with another QTL/m-QTL associated with Zn accumulation was observed. MTP11 was also found to be overlapping with QTL associated with Cd tolerance (Table 4).

**Table 4:** Presence of MTP genes in various rice QTLs/meta-QTLs associated with metal tolerance/accumulation.

QTL/Meta-QTL	Associated Metal	Chromosome No.	Associated Markers	QTL/Meta-QTL position (bp)	Gene within QTL/Meta-QTL	Reference
rMQTL3.2	Zn	3	RM7425	6,164,117–8,406,578	*MTP8*.*1*	Jin et al. 2015
rMQTL3.1	Zn	3	RM489	2,454,089–7,233,990	*MTP8*.*1*	Jin et al. 2015
rMQTL5.1	Zn	5	RM3437	8,304,202–19,608,342	*MTP1*	Jin et al. 2015
qSh4b	Zn	4	None	16.38–16.87 Mb	*MTP9*	Zhang et al. 2017
*qRL1*.*3*	*Cd*	1	C225-RG536	41450113–41451272, 22339310–37713775	*MTP11*	Xue et al. 2008

### Gene expression dynamics of MTP genes during vegetative growth

**Fig 3:**
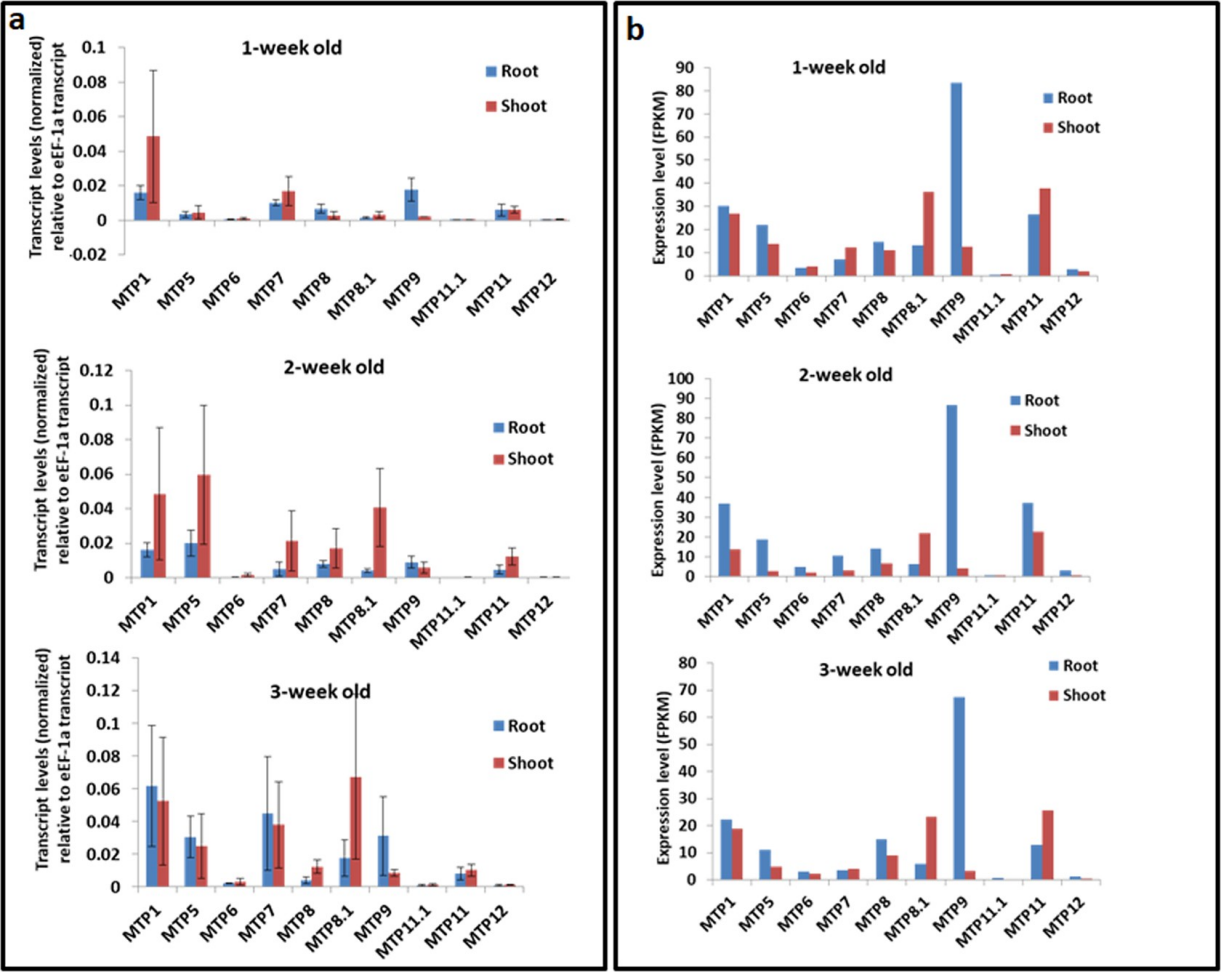
Expression profiling of rice metal tolerance protein (MTP) genes in root and shoot tissues of 1-week, 2-week, 3-week old seedlings. (a) Q-PCR analysis of MTP transcripts. Transcript levels of the MTP genes were normalized with transcript levels of internal reference gene *OsUBQ5*. Then the resulting expression level of MTP genes was compared to normalized expression level of constitutively expressed *OseEF-1a* gene. The resulting levels of expression of MTPs are compared among themselves as well as between roots and shoots. (b) Expression values in FPKM (fragments per kilobases of gene per million mapped reads) obtained from Rice Expression Database (http://expression.ic4r.org/).

To examine the expression pattern of MTP genes during the vegetative phase, we analyzed their expression separately in root and shoot tissues in one-week, two-week and three-week-old rice seedlings through Quantitative-PCR **(**Q-PCR) (Fig 3A). Additionally, we also examined the expression pattern of MTP genes in public transcriptomic data available at Rice Expression Database (Fig 3B). The Rice Expression Database hosts most of the publicly available RNA-Seq datasets for rice, with normalized gene expression in the form of FPKM (fragments per kilo-base of gene per million mapped reads) values [21]. Significantly, we have found an excellent correlation between our Q-PCR analysis and public transcriptomic data, (Fig 3A and 3B). For instance, in both Q-PCR and public transcriptome data, expressions of MTP6, MTP11.1 and MTP12 was found lowest among all MTPs under tested conditions. Similarly, MTP9 showed root specificity during all the three-development stages in both the datasets (Fig 3A and 3B). Furthermore, MTP8 was found to have intermediate expression levels among all MTPs in both root and shoot tissues of all three developmental stages in both Q-PCR and public transcriptomic data (Fig 3A and 3B). There are some examples in which we found differences between Q-PCR and public transcriptomic data. For example, MTP1 and MTP7 are consistently expressed at all these developmental stages at the highest levels in our Q-PCR analysis, but in public transcriptomic data only MTP1 was expressed at the highest level (Fig 3A and 3B). To understand temporal patterns of expression, comparison of gene expression was performed between different developmental stages. Some MTP genes, like MTP5 and MTP8.1, have the least expression at one-week stages in both root and shoot tissues, however at later stages (two- and three-week) their expression increases in both the tissues (Fig 3A). MTP8.1 shows shoot specific expressions in all the three development stages in public transcriptomic data, and only at 2-week-old stage in our Q-PCR analysis (Fig 3A and 3B). In conclusion, combining Q-PCR and public transcriptomics results provides a detailed description of the expression dynamics of MTP genes during vegetative stages. To gain insight about relative expression levels of MTP genes in these tissues through Q-PCR experiments, we have calculated expression levels of MTP genes with relation to expression of OseEF-1a gene (Fig 3A). As shown in Fig 3A, the expression of MTP genes in descending order in roots was MTP9>MTP1>MTP7>MTP8>MTP11>MTP5>MTP8.1>MTP6> MTP12>MTP11.1. In the shoots a similar pattern was observed.

### Expression profiling of rice MTP genes during seed development

**Fig 4:**
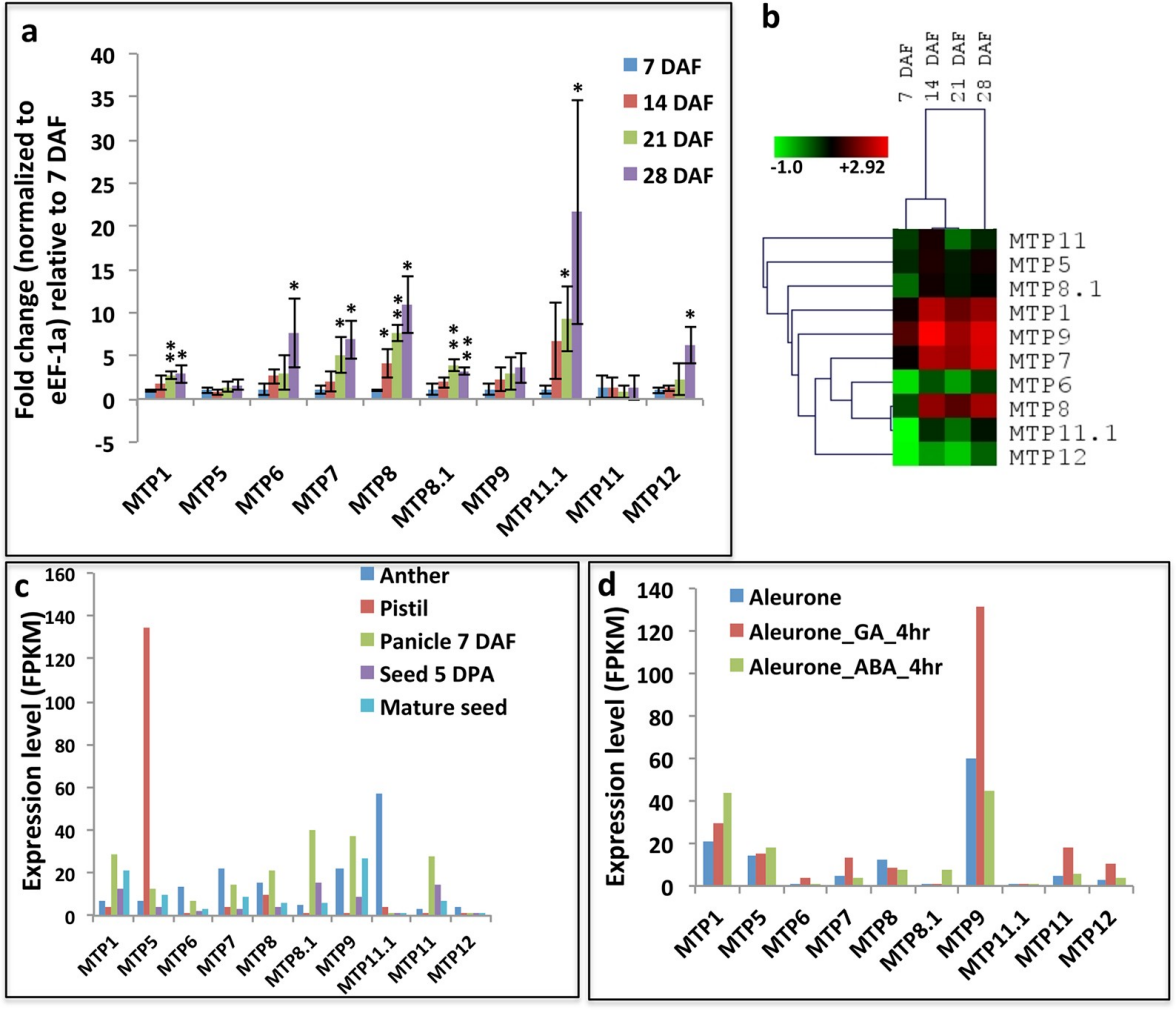
Expression profile of rice MTP genes during reproductive growth. (**a**) Q-PCR analysis showing normalized expression of MTP genes in developing seeds at different DAF (Days After Fertilization) stages. Fold change between different time points is calculated against 7 DAF stage. Asterisks indicate T-test results where * = p<0.05, ** = p<0.01, *** = p<0.001. (**b**) Hierarchical clustering of Q-PCR results showing the expression profile of MTP genes in developing seeds. Relative fold change of MTP expression (normalized against *OsUBQ5*) was calculated against *OseEF-1a* expression (normalized against *OsUBQ5*) gene. The resuting fold change values were then used to make heatmap. The heatmap representing hierarchical clustering of log2 transformed expression values of MTP genes at different developmental stages (indicated at the top of each lane) was generated using MeV software package (http://mev.tm4.org/). The color bar below represents relative expression value from green color representing the lowest expression levels, black represents medium expression levels, and red signifies the highest expression level. (**c, d**) Expression values of rice MTP genes at various stages of reproductive growth shown as FPKM (fragments per kilobases of gene per million mapped reads) values obtained from Rice Expression Database (http://expression.ic4r.org/). (**d**) Expression in aleurone layer and effect of GA (Gibberellic Acid) and ABA (Abscisic Acid) on aleuronic expression.

No previous study has examined the expression dynamics of MTP genes during seed development. Hence, to understand the expression pattern of rice MTP genes during seed development, Q-PCR analysis was performed on developing seeds at 7, 14, 21 and 28 Days After Flowering (DAF) stages (Fig 4A and 4B). In addition, the expression pattern of MTP genes was analyzed in different reproductive tissues and seed tissues in the public transcriptomic data (Fig 4C and 4D).

The Q-PCR analysis showed expression of most of the MTP genes increased as the seed development progressed, except for expression of MTP5, MTP9 and MTP11 (Fig 4A). Consistently, in the public transcriptomic data the expression of most MTP genes was higher in mature seed compared to 5 DPA (Day Post Anthesis) old seed (Fig 4C). These results suggest that MTP genes are likely to play an important role in filling metal micronutrients in developing seeds. When expression of MTP genes was compared among themselves during seed development, we found that MTP9 has maximum expression in all the four analyzed seed development stages, followed by MTP1, MTP7 and MTP8 (Fig 4B). This observation was also partially true with public transcriptomics data (Fig 4C). Expression of other MTP genes was found minimal during different stages of seed development (Fig 4B). Notably, in the public transcriptomics data, we found that MTP5 specifically expresses in pistil and MTP11.1 specifically in anthers (Fig 4C), which suggests their potential role in these tissues. Furthermore, we note that in the public transcriptomics data MTP9 is strongly expressed in aleurone layer, and up-regulated by Gibberellic Acid (GA) treatment (Fig 4D), which is consistent with maximum expression of this gene in seed tissue among all MTPs. Overall, our results identify reproductive tissue and stage specific MTP genes.

### Effects of heavy metal stress on MTP gene expression

Since previous studies highlight the role of few MTP genes in tolerance to heavy metal stress [22,23], so we decided to thoroughly investigate the effect of different heavy metals on expression of all the MTP genes. To impose heavy metal stress, rice seedlings were transferred to hydroponics media containing different heavy metals for different time points. Subsequently, root and shoot tissue were collected separately and analyzed for gene expression through Q-PCR. There was no obvious effect on plant growth with 24 hour (hr) treatment of Cobalt (Co), Cadmium (Cd) and Nickel (Ni). However, after one week of treatment expression of most MTP genes was down-regulated in both root and shoot tissues (S1 Fig). This suggests that longer heavy metal treatments have altered plant physiology, so we will mainly discuss our gene expression results for only 24 hours of heavy metal treatment.

**Fig 5:**
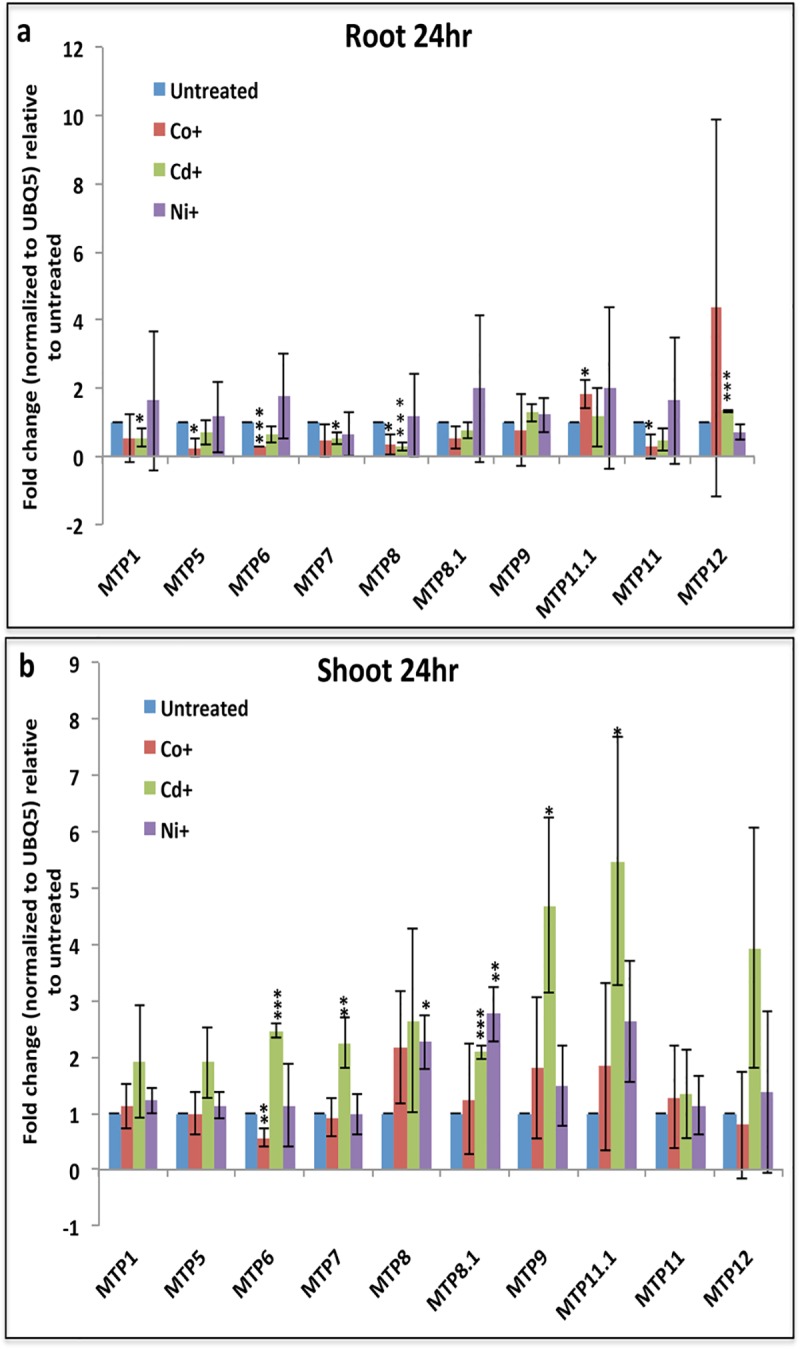
Q-PCR analysis of gene expression changes of rice MTP genes in response to various heavy metal stress conditions. Q-PCR analysis of MTP genes in roots (a), and shoots (b), in response to 24-hour exposure to Co, Cd, and Ni. The fold change is calculated against untreated samples. Asterisks indicate Student’s T-test results, where * = p<0.05, ** = p<0.01, *** = p<0.001.

At 24 hours of treatment, most of the MTP genes showed gene expression changes either in root or shoot (Fig 5). Interestingly, we found that among the differentially expressed genes, most genes show down-regulation in the roots (Fig 5A), and up-regulation in the shoot (Fig 5B). The exception to this notion was MTP6, which was repressed by Co in both root and shoot tissues (Fig 5). Furthermore, it was found that most genes were affected only with one out of three different heavy metal stress conditions (Fig 5), suggesting that these genes have specificity towards particular heavy metal species. The few notable exceptions to this notion includes MTP8 which shows down-regulation in roots by both Co and Cd, MTP8.1 which shows up-regulation by both Cd and Ni in shoot, and MTP6 which show down regulation by Co and up-regulation by Cd in shoot (Fig 5), suggesting that these genes have broad sensitivity towards different heavy metal species. Meticulously, in roots, only Cd and Co were found to be affecting the expression of MTP genes, as expression of none of the MTP genes was found to be affected by Ni treatment (Fig 5A). However, in the shoot we found that Cd affects the most number of the MTP genes (Fig 5B).

### Effects of zinc and iron starvation conditions on MTP gene expression

**Fig 6:**
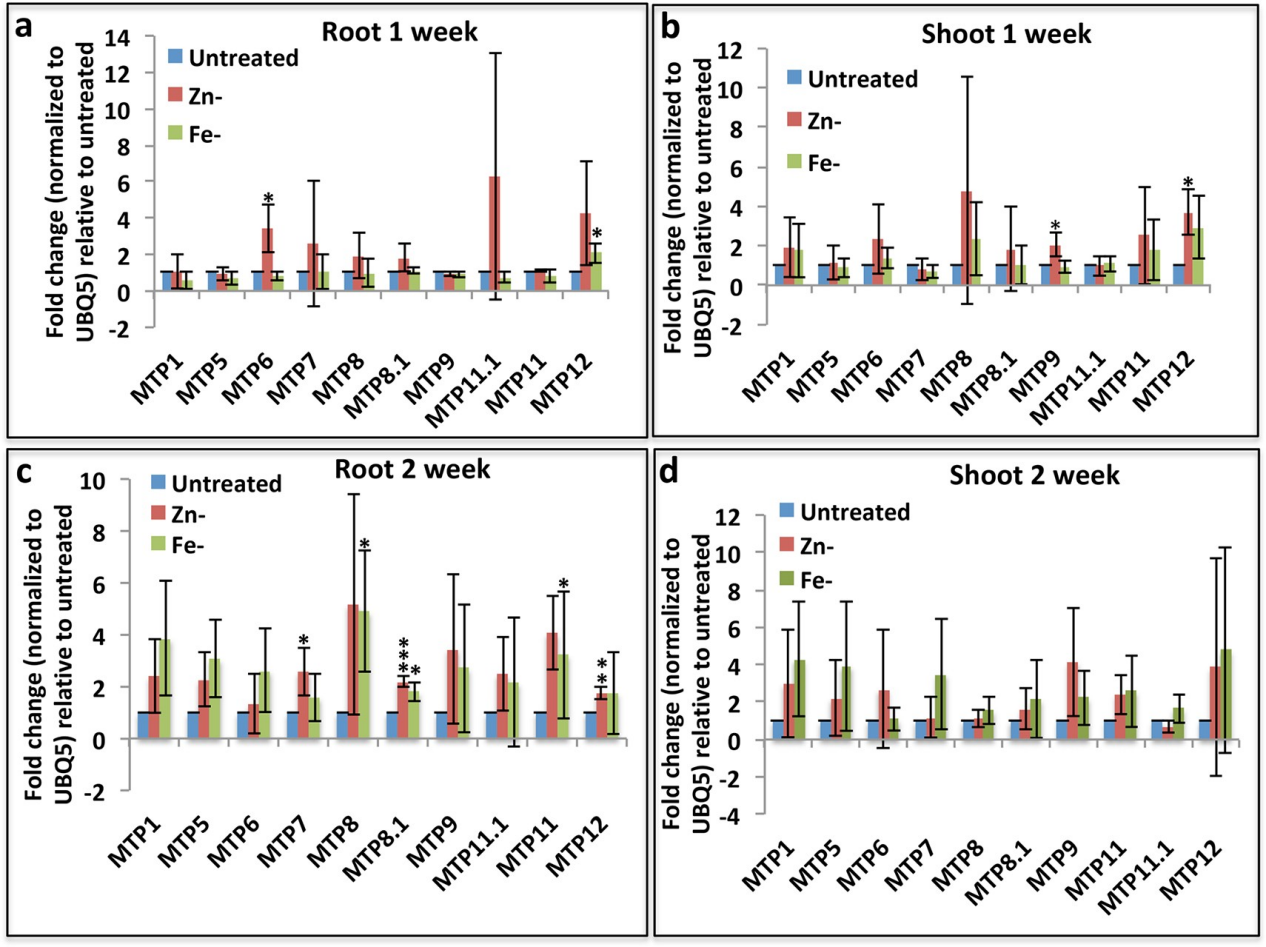
Gene expression changes of rice MTP genes in response to starvation conditions of Zn and Fe. Q-PCR analysis of MTP genes in roots (a, c) and shoots (b, d) in response to 1 week (a, b) or 2 week (c, d) of Zn or Fe deficiency conditions. The fold change is calculated against untreated samples. Asterisks indicate T-test results where * = p<0.05, ** = p<0.01, *** = p<0.001.

Since some of the plant MTP genes are known to transport essential heavy metals, such as Fe and Zn [24], so we asked which rice MTP genes are likely to have this function. For this purpose, we exposed the rice seedlings to media depleted with these mineral elements and performed gene expression analysis through Q-PCR. Contrary to mis-expression of most MTP genes by non-essential heavy metals, expression of only a handful of MTPs genes was found to be affected by one week Zn and Fe starvation conditions (Figs 5 and 6). For instance, expression of only MTP6 (in Zn-) and MTP12 (in Fe-) was up-regulated in roots after 1 week of starvation (Fig 6A). However, after 2 weeks of starvation, we found that expression of MTP7, MTP11, and MTP12 (all in Zn-), MTP8 (in Fe-), and MTP8.1 (in both Zn- and Fe-) genes were also significantly up regulated in roots (Fig 6C). These results suggest the potential role of these genes in the transport of Fe and Zn in roots. In the shoots, MTP9 and MTP12 were found to be significantly up-regulated at 1 week of Zn deficiency conditions (Fig 6B). Significantly MTP9 was also found to be highly expressed in seed aleurone layer, and the aleurone layer accumulates very high levels of Zn and Fe (Fig 4D). Altogether these results very strongly suggest that MTP9 might be a good target for future Zn biofortification strategies.

## Discussion

Plants have evolved several molecular mechanisms involving specific gene families to tolerate toxic levels of heavy metals. Considerable work has been done to understand the function of plant MTP/CDF gene family in heavy metal stress tolerance. In rice, MTP genes were identified and named after completion of genome sequencing, based on the sequence similarity with Arabidopsis MTP genes[11, 12]. Previous studies mainly looked at phylogenetic relationships among various CDFs across diverse taxa and classification of plant MTPs into various groups based on substrate specificity. Other more recent studies were aimed at functional analysis of individual rice MTP genes [14, 15, 24]. The present study is an attempt to comprehensively understand the evolution of rice MTP genes and their response to essential and non-essential heavy metals.

Gene intron-exon organizations have been considered as more of an efficient model to study evolutionary relations between orthologues and paralogues. Comparison of intron-exon organization with respective phylogenetic distribution showed similar pattern for the phylogenetically closer MTP genes (Fig 1A). In addition, numbers of introns in the gene have been reported to correlate with gene expression, and loss or gain of homologues. Mostly, intronless genes in plants are thought to have evolved recently [25]. Among the rice MTPs, intron-less MTP1 and MTP12 genes do not show any other characteristic (Tajima’s D and SNPs) suggesting early evolution. Interestingly, MTP1 and MTP12 share a characteristic histidine rich loop towards c-terminal, which is known to have a role in Zn-binding. The phylogenetic distribution and gene intron-exon structure have been helpful in understanding the evolutionary relationship of MTP genes.

The whole genome re-sequencing information available for 3000 diverse rice genotypes is an excellent resource for the identification of allelic diversity and to study evolution. Wide range of allelic diversity for the MTP genes observed in the present study will be helpful in exploring the genetic resources for the development of heavy metal tolerant varieties as well as varieties with efficient uptake of essential elements. The rare allele of MTP8.1 with three amino acid changes compared to the most of the japonica cultivars provide an opportunity to evaluate functional impact and subsequent exploration through breeding approaches (Table 5). The amino acid change from A to S predicted in MTP8.1 to have a deleterious effect suggesting significant variation in the functionality of the alleles. The results look promising in view of the previous study performed in soybean, where the effects predicted with PROVEAN tool have been validated [26]. Analysis of the functional effect of different alleles of MTP genes is beyond the scope of present study but the information presented here will serve for the study aiming to explore genetic resources. The evolutionary parameters studies here suggest balancing evolution for MTP genes in rice. Earlier similar type of selection pressure suggesting balancing evolution instead of selective sweeps have been reported for the R genes [27].

**Table 5:** Functional effect prediction for the amino acid changes located in the candidate OsMTP8 gene. Neutral or deleterious effect was predicted based on the score estimated using PROVEAN tool (http://provean.jcvi.org/about.php).

Gene_ID	Protein_position	Amino_acids	Codons	Score	Prediction (cutoff = -2.5)
LOC_Os03g12530	54	S/F	tCc/tTc	-0.712	Neutral
	**76**	**E/A**	**gAg/gCg**	**-2.692**	**Deleterious**
	144	G/S	Ggt/Agt	-1.078	Neutral
	276	A/S	**G**cc/**T**cc	-1.156	Neutral

Expression pattern of a gene can give clues about its function. In the present study, we have analyzed the expression profile of all the MTP genes across various tissues and developmental stages through Q-PCR and in transcriptomics data available in the public domain. Most significantly, we have found a good correlation between these two independent analyses, which gives very high confidence about these results. Since the expression pattern of some of the rice MTPs was previously analyzed [14, 15, 24], so we compared our results with these studies and found that our gene expression results are comparable to them. For example, Yuan *et al*. [23] found that MTP1 has higher expression in two-week old leaves compared to roots, our Q-PCR results also confirm this (Fig 3A). Zhnag *et al*. [16] and Chen *et al*. [14] found a higher expression of MTP8 and MTP8.1, respectively, in leaves of two-week old seedlings compared to their roots, our Q-PCR results also confirm this notion (Fig 3A). In conclusion, some of our findings have been confirmatory in nature in cases where the individual MTP gene has been previously characterized. However, for other MTP genes, which are not yet functionally characterized, our results provide novel insights about their expression patterns in different tissues and organs.

Expression analysis for any of the rice MTP genes in seed and reproductive tissues has not been performed earlier. Expression profile analysis derived from transcriptome data and Q-PCR analysis revealed that the tissue expression patterns of OsMTPs in the same group were similar but varied among groups (Fig 3). Similar results have been obtained in current study performed for MTP genes in *Nicotiana tabacum* [28]. Together these results indicate the conserved and essential roles of MTP genes across various growth and development stages of various plants. Analysis of expression values in public transcriptomics data revealed anther and pistil specific expression of some of the MTP genes (Fig 4C), which was not reported earlier. Furthermore, as the seed development progressed, expression of most of the MTP genes was also found to be increased (Fig 4A). As seeds are major sink organ and sink strength of the seeds increases along with their development [29], so increased expression of MTP genes during seed development indicates their potential role in metal homeostasis during seed filling stage. Borill *et al*. [30] have proposed potential role of MTP genes in accumulation of essential heavy metals in vacuoles of aleurone layer. Supporting this, our results indicate that MTP9 is strongly expressed in aleurone layer (Fig 4D). Very recently it has been shown that MTP9 expresses in cell-type specific manner in rice roots, where it expresses only in exo- and endodermis cells of the roots [15]. This further supports the role of MTP9 as a cell-type specific player. Ueno *et al*. [15] also reported 40% yield loss and 50% reduction in total seed Mn content in brown rice in knock-down lines of MTP9 genes, which strongly support its role in essential metal filling in developing seeds. However, it was not analyzed if there were reduction in accumulation of other essential metals such as Zn and Fe.

In order to understand the response of MTP genes to heavy metals, we analyzed their gene expression either in the absence of essential heavy metals Zn and Fe or in the presence of non-essential heavy metals such as Cd, Co and Ni. Expression of most MTPs is increased in shoot and decreased in roots after 24 hr treatment with non-essential heavy metals (Fig 5). These results suggest a potential heavy metal tolerance mechanism in which plants accumulate heavy metals away from their site of exposure (roots in this case). Hyper-accumulator plants use the same strategy to accumulate heavy metals in their aboveground parts [10].

In comparison to expression of most of the MTP genes affected by non-essential heavy metals, the expression of only a few MTP genes was affected by Zn and Fe deficiency (Figs 5 and 6). Furthermore, the expression of the affected MTP genes was induced in both roots and shoots by deficiency of both Zn and Fe, which suggests that both roots and shoots induce the mechanism of sequestration. Zn deficiencies in the early stages of seedling development and iron deficiency chlorosis of leaves are two major growth challenges for rice seedlings [30, 31]. A proper understanding of homeostasis of these ions in seedlings stages would help to design crops with better survival under nutrient deficient soil.

## Supporting information

S1 Fig Q-PCR analysis of gene expression changes of rice MTP genes in response to 1 week exposure to various heavy metal stress conditions. Q-PCR analysis of MTP genes in roots (**a**), and shoots (**b**), in response to one-week exposure to Co, Cd, and Ni. The fold change is calculated against untreated samples. Asterisks indicate Student’s T-test results, where * = p<0.05, ** = p<0.01, *** = p<0.001.
(PDF)

S1 Table Protein structure and localization of rice MTP proteins. (DOCX)

S2 Table Primer sequence used for Q-PCR. (DOCX)

S3 Table Raw data and normalized expression values for all the Q-PCR experiments. (XLSX)
