# Supplementary figures and images for: Molecular characterization and expression dynamics of MTP genes under various spatio-temporal stages and metal stress conditions in rice
Source: PLoS One. 2019 May 28;14(5):e0217360. doi: 10.1371/journal.pone.0217360 (PMC6538162; doi:10.1371/journal.pone.0217360)

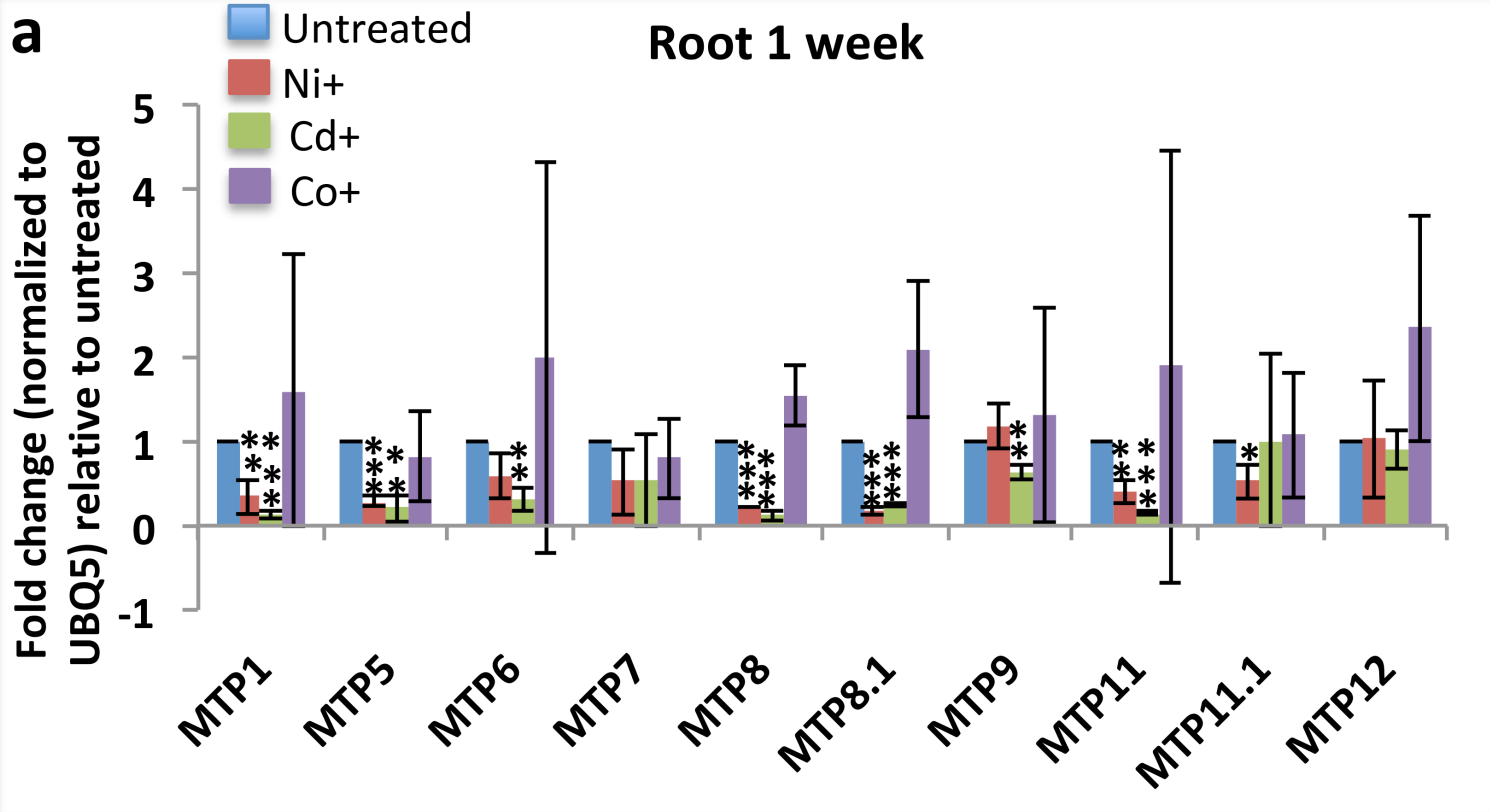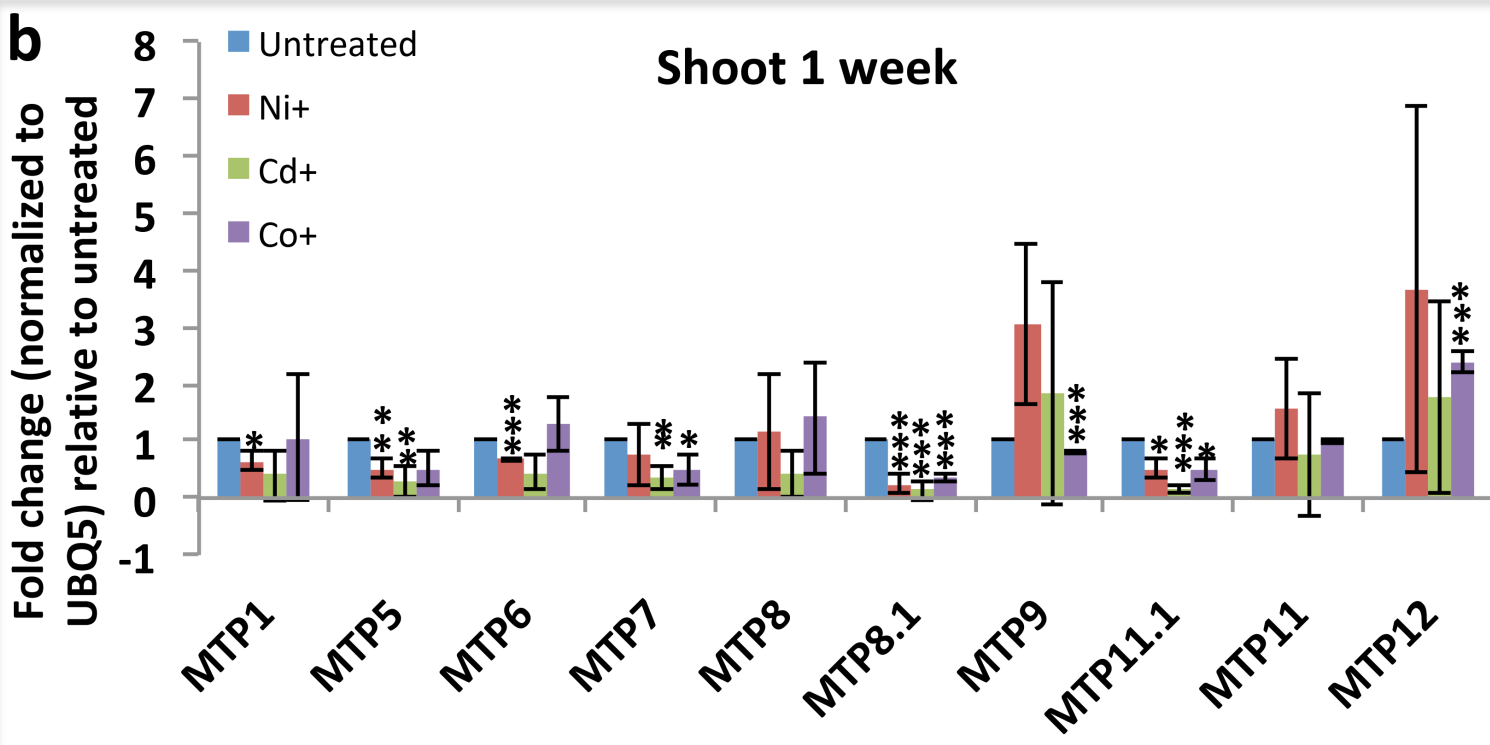

Supplement: S1 Fig — Q-PCR analysis of MTP genes in roots (a), and shoots (b), in response to one-week exposure to Co, Cd, and Ni. The fold change is calculated against untreated samples. Asterisks indicate Student’s T-test results, where * = p<0.05, ** = p<0.01, *** = p<0.001. (PDF) [file pone.0217360.s001.pdf]
