# Supplementary material for: Molecular characterization and expression dynamics of MTP genes under various spatio-temporal stages and metal stress conditions in rice
Source: PLoS One. 2019 May 28;14(5):e0217360. doi: 10.1371/journal.pone.0217360 (PMC6538162; doi:10.1371/journal.pone.0217360)
Supplement: S1 Table — (DOCX) [file pone.0217360.s002.docx]

| **S. No.** | **Gene ID** | **Length** |  | **TMHMM^1^** |  | **CELLO Server^2^** |
| --- | --- | --- | --- | --- | --- | --- |
|  |  |  | **ExpAA** | **First60** | **TMH** |  |
| 1 | *OsMTP1* | 418 | 130.75 | 3.14 | 6 | Plasma Membrane |
| 2 | *OsMTP5* | 276 | 95.39 | 17.23 | 5 | Plasma Membrane |
| 3 | *OsMTP6* | 509 | 38.01 | 0.01 | 0 | Mitochondria |
| 4 | *OsMTP7* | 472 | 89.62 | 0 | 4 | Chloroplast |
| 5 | *OsMTP8* | 410 | 113.14 | 0 | 5 | Plasma Membrane |
| 6 | *OsMTP8.1* | 397 | 106.6 | 0 | 5 | Plasma Membrane |
| 7 | *OsMTP9* | 391 | 115.57 | 0 | 6 | Plasma Membrane |
| 8 | *OsMTP11* | 415 | 96.71 | 0.25 | 4 | Plasma Membrane |
| 9 | *OsMTP11.1* | 376 | 66.84 | 0 | 3 | Plasma Membrane |
| 10 | *OsMTP12* | 800 | 269.89 | 10.28 | 10 | Plasma Membrane |

**Table 4.** Protein structure and localization of rice MTP proteins

^1^http://www.cbs.dtu.dk/services/TMHMM/; ^2^http://cello.life.nctu.edu.tw/
