# Supplementary material for: Molecular characterization and expression dynamics of MTP genes under various spatio-temporal stages and metal stress conditions in rice
Source: PLoS One. 2019 May 28;14(5):e0217360. doi: 10.1371/journal.pone.0217360 (PMC6538162; doi:10.1371/journal.pone.0217360)
Supplement: S2 Table — (DOCX) [file pone.0217360.s003.docx]

| Primer Name | Sequence (5' to 3') |
| --- | --- |
| OsMTP11 (Os01g0837800) F RT | CACGCTCTGTCCCATGAAAA |
| OsMTP11 (Os01g0837800) R RT | GTTTCCTGACGAGCACCAG |
| OsMTP8.1 (Os03g0226400) F RT | CCAACCATGACCACCAACTT |
| OsMTP8.1 (Os03g0226400) R RT | GCACGTACTGTAGCACATCC |
| OsMTP9 (Os01g0130000) F RT | AGGCCGAGGTCTGAATTCTC |
| OsMTP9 (Os01g0130000) R RT | CACAGGGAAACGATGACTTGT |
| OsMTP1 (Os05g0128400) F RT | CATTCAGATTGAGCGCGAGT |
| OsMTP1 (Os05g0128400) R RT | AGTTGGTCCCATGAGATGAGA |
| OsMTP5 (Os02g0832700) F RT | ACCTGACCATCCAAACCGAT |
| OsMTP5 (Os02g0832700) R RT | ACTGCGAGAGTTTGATCTATCCT |
| OsMTP6 (Os03g0346800 ) F RT | TGGGCCAGCAAATTAAGCAA |
| OsMTP6 (Os03g0346800 ) R RT | TCAACCGTGACAGTGTAGCT |
| OsMTP7 (Os04g0298200) F RT | TGAAGCACACAACCCAGAAG |
| OsMTP7 (Os04g0298200) R RT | ACAGCAAGTGAGACCAATCG |
| OsMTP8 (Os02g0775100) F RT | GCGTGCTTTTGTTCATGTGG |
| OsMTP8 (Os02g0775100) R RT | TGCACAAGAGTAAAACTGGAGC |
| OsMTP11.1 (Os05g0461900) F RT | AGCTTACCTACCTGTGCTGG |
| OsMTP11.1 (Os05g0461900) R RT | ACAAAGTAGTGGGAGCCGAA |
| OsMTP12 (Os08g0422200) F RT | GGCACTTTTCACCTTCATATCAC |
| OsMTP12 (Os08g0422200) R RT | TCTTGAATCCCAGCCTCATGA |
| Ubiquitin 5 F RT | ACCACTTCGACCGCCACTACT- |
| Ubiquitin 5 R RT | ACGCCTAAGCCTGCTGGTT |
| eEF-1a F RT | TTTCACTCTTGGTGTGAAGCAGAT |
| eEF-1a R RT | GACTTCCTTCACGATTTCATCGTAA |

**S1 Table. Primer sequence used for Q-PCR**
